# Editorial: The Role of the IGF/Insulin-IGFBP Axis in Normal Physiology and Disease

**DOI:** 10.3389/fendo.2022.892140

**Published:** 2022-04-21

**Authors:** Shin-Ichiro Takahashi, Claire M. Perks

**Affiliations:** ^1^ Graduate School of Agriculture and Life Sciences, The University of Tokyo, Tokyo, Japan; ^2^ Department of Translational Health Sciences, University of Bristol, Bristol, United Kingdom

**Keywords:** IGF, IGFBPs, insulin, ageing, cancer, GRC

The 2019 Gordon Research Conference (GRC) on the insulin-like growth factor (IGF) and Insulin System in Physiology and Disease was held from March 10th to 15th in 2019. In that meeting, we focused on “The Impact of IGF and Insulin on Life-Long Health”. We discussed cutting-edge research on the fundamental roles of IGF and insulin in normal physiology and diseases particularly related to aging, cancer, and metabolic disorders. IGFs and insulin are conserved throughout evolution to mediate the effects of nutrition on growth, metabolism, and development, and hence play a significant role in health and disease over the lifespan. They modulate diverse aspects of cell function such as proliferation, differentiation, survival, and metabolism of most physiological systems in the body. The IGF family of ligands, receptors, and IGF binding proteins are frequently affected in many pathological conditions, such as growth failure, diabetes, cancer, and degenerative diseases, and therefore have become attractive therapeutic targets. This conference encompassed ground-breaking information regarding critical characteristics of the biology of the IGF/insulin family in both normal physiology and pathological states highlighting innovative methodologies and novel interactions (*e.g.*, stem cell biology and the microbiome). We selected eight exciting topics presented at the GRC for this special issue on “The Role of the IGF/Insulin-IGFBP Axis in Normal Physiology and Disease” The IGFBPs are frequently dysregulated in pathological conditions and Duan and Allard, discussed what is currently known about IGFBP-5 in normal physiology and human disease. They concluded that IGFBP-5 is a multifunctional protein that can act as a molecular switch to regulate IGF signaling conditionally. Therapy resistance is a major problem in cancer treatment and Zheng et al., discovered that IGFBP-1 plays a significant role in resistance to a selective estrogen receptor modulator and antagonist for estrogen receptor alpha (ERα) in breast tissue, called Tamoxifen. IGFBPs can be post-translationally modified, for example *via* proteolytic cleavage and this also has implications for disease. Hoeflich et al., found that reduced fragmentation of IGFBPs and concomitant reduction of IGF-II to IGFBP ratios modulated the bioactivity of IGF-II in cerebrospinal fluid during repeated intrathecal triamcinolone acetonide administration in multiple sclerosis patients, which may have relevance for treatment. In addition, Hjortebjerg et al., showed that pregnancy-associated plasma protein-A (PAPP-A) and its homolog PAPP-A2 which are reported as IGFBP proteases are enzymes that modulate the availability and mitogenic activity of IGF-I. Collectively, the data show that PAPP-A2, but not PAPP-A, is elevated in patients with lung cancer and is associated with mortality. This novel role of PAPP-A2 in cancer warrants further functional studies as well as validation in external cohorts. As for signal transduction of insulin-like peptides, the mini-review by Rieger and O’Connor, introduced data showing that IGF-I receptor endocytosis and trafficking to specific subcellular locations can define specific signaling responses that are important for key biological processes in normal cells and cancer cells. Once internalized, the IGF-I receptor may be recycled, degraded, or translocated to the intracellular membrane compartments of the Golgi apparatus or the nucleus leading to different outcomes. Okino et al., showed that the high levels of insulin receptor substrate (IRS)-1 in myoblasts induces their elimination from the cell layer due to abnormal sustainment of IGF-I receptor activation. This cell competition plays a vital role in myotube formation. The mini-review by Barker et al., presents a brief overview examining aspects of IGFs and the PI3K/Akt pathway in two apparently unconnected diseases: Alzheimer’s dementia and cancer. Although these disease states appear to be opposed, the same vital molecules are controlling pathology and, differential targeting of therapeutics, may benefit both. Finally, Stuard et al., provided the latest update on the function of IGF and related proteins in corneal development, during wound healing, and in the pathophysiology of disease and highlighted key areas of research that are necessary for future studies. From *C. elegans* to rhesus monkeys, it has been reported that suppression of insulin-like activity is associated with an increased life span. Together with other reports from this GRC, excessive induction of insulin-like activity can lead to cancer, and excessive attenuation leads to various diseases that are also problematic in an aging society, such as Alzheimers. These results clearly demonstrate the importance of regulating insulin-like activity to an appropriate range to maintain a lifetime of good health. This regulation is accomplished through ligand production, interaction with binding proteins, receptor expression, and signaling. The future mission of this research area is to elucidate how abnormalities in molecular signalling pathways utilized by the IGF axis correlate with the phenotype associated with pathological conditions, to develop preventive and therapeutic interventions, leading to higher quality resource animals and an increase in healthy life expectancy in humans. The IGFs are clearly relevant to all life phenomena and as such have attracted many researchers to the field from different backgrounds, highlighted by the varied and diverse presentations covering both normal physiology and disease. An IGF focus within a cross-disciplinary approach yields exciting, novel and groundbreaking discoveries that were presented at the GRC, and the participants all shared in the excitement. We are delighted for the broader community to provide a taste of this GRC in this special issue. The next GRC on the IGF and Insulin System in Physiology and Disease will be held in March 2023. We look forward to seeing you all there.

## Author Contributions

ST and CP co-wrote the editorial. All authors contributed to the article and approved the submitted version.

## Funding

This work was supported in part by Grants-in-Aid for Scientific Research (A) #18H03972, Advanced Research Networks from the Japan Society for the Promotion of Science (JSPS) and R&D matching funds on the field for Knowledge Integration and innovation and Moonshot Agriculture, Forestry and Fisheries Research and Development Program from Bio-oriented Technology Research Advancement Institution (BRAIN) to ST.

## Conflict of Interest

The authors declare that the research was conducted in the absence of any commercial or financial relationships that could be construed as a potential conflict of interest.

## Publisher’s Note

All claims expressed in this article are solely those of the authors and do not necessarily represent those of their affiliated organizations, or those of the publisher, the editors and the reviewers. Any product that may be evaluated in this article, or claim that may be made by its manufacturer, is not guaranteed or endorsed by the publisher.

